# Supplementary material for: A SUPERMAN-like Gene Controls the Locule Number of Tomato Fruit
Source: Plants (Basel). 2023 Sep 21;12(18):3341. doi: 10.3390/plants12183341 (PMC10535046; doi:10.3390/plants12183341)
Supplement: Supplementary file 1 [file plants-12-03341-s001.zip › plants-2618626-supplementary.pdf]

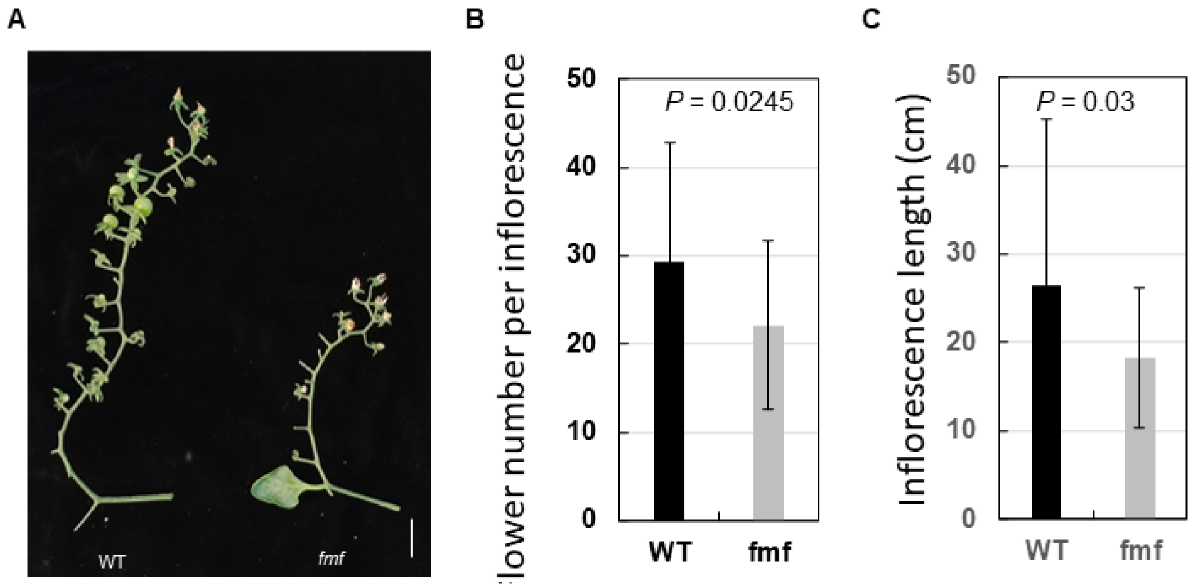

Figure S1. Inflorescence phenotype of *fmf* and wild type.

A, inflorescences of wild type and *fmf*. **B**, flower number per inflorescence of wild type and *fmf* plants. **C**, length of wild type and *fmf* inflorescences. The 3rd to 5th inflorescences from 10 wild type and 10 *fmf* plants were measured, respectively. Data represent means ±SD. *P* values were calculated using Student's t-test. Scale bar, 1 cm.

A

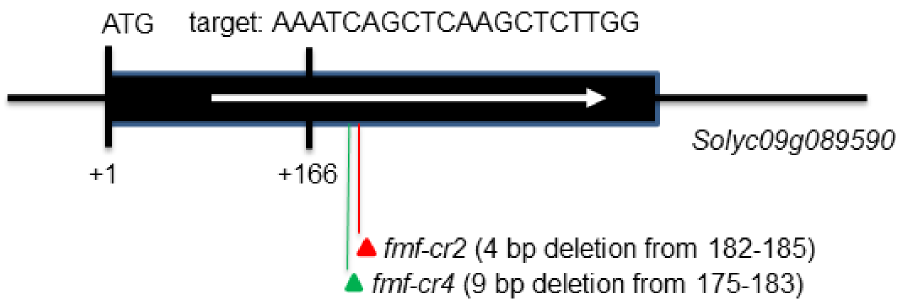


```
fmf-cr2:
MEKNSSKYFNKHQTMKNVKEYSWDNNYVDHQGGDLVGGFLWPPRSYTCSFCKR
EFKSAQALVI*
fmf-cr4:
MEKNSSKYFNKHQTMKNVKEYSWDNNYVDHQGGDLVGGFLWPPRSYTCSFCKR
EFKSA---GGHMNVHRRDRARLRLQSPTILESNPNPNSNPNPNPSFVSSPSSP
STKLFPPFVSTLPPLLSPNSFSSSTAAGGGSHEMKNGDLTKMGSAKFEENGKE
CSEVVKRSEFLRLDLGIGLISESKDDLDLDLELRLGYI
WT:
MEKNSSKYFNKHQTMKNVKEYSWDNNYVDHQGGDLVGGFLWPPRSYTCSFCKR
EFKSAQALGGHMNVHRRDRARLRLQSPTILESNPNPNSNPNPNPSFVSSPSSP
STKLFPPFVSTLPPLLSPNSFSSSTAAGGGSHEMKNGDLTKMGSAKFEENGKE
CSEVVKRSEFLRLDLGIGLISESKDDLDLDLELRLGYI
```

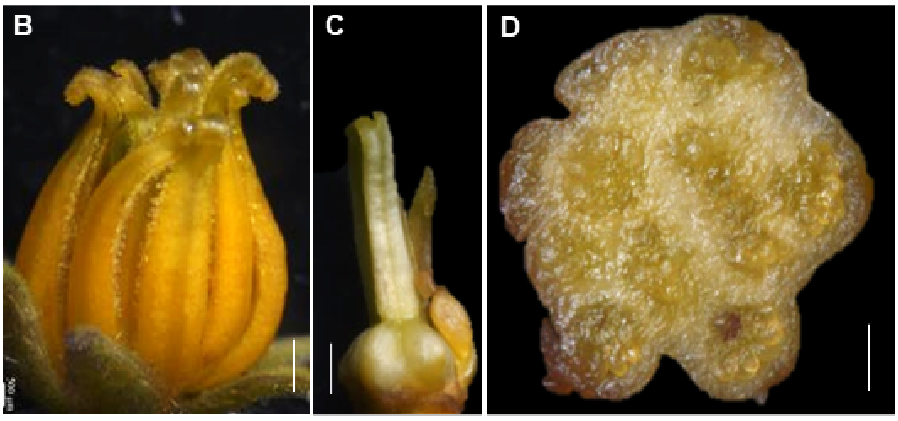

Figure S2. Generations of *fmf* mutants by CRISPR-CAS9.

A, generation of *fmf* mutants by gene editing. Two different alleles were obtained. The two alleles show identical phenotypes, which *fmf-cr2* likely encodes truncated protein and *fmf-cr4* encodes a protein missing three amino acids (QAL) in the conserved C_2H_2 domain. The *fmf-cr2* phenotypes was shown in **Figure 6**. **B-D,** images showing abnormal floral phenotypes of the *fmf-cr4* mutant in Moneymaker background. Scale bar, 500 μm.

Table S1. Primers for DNA markers used in this study.

Primers	chr	Sequences (5'--3')	pair	Tm	Chromosome position	Fragment size
XPS2448F	9	TCCTATTTTGCTAGTTTGTGTACA	XPS2448R	56.3	SL4.0ch09:64554696..64554845,64554726	86bp/150bp
XPS2448R	9	AAGAGTTGTAACATGCACGA		55.3		
XPS2450F	9	CTGTTTCATGTCATCTCTCTTGA	XPS2450R	56.3	SL4.0ch09:62245187..62245362,62245257	176bp/135bp
XPS2450R	9	ACCAACTTCAGTCGCTCTTT		57.4		
XPS2451F	9	GATGCGTTTAGATTCGAAGGTT	XPS2451R	57.1	SL4.0ch09:63284970..63285196,63285092	227bp/170bp
XPS2451R	9	AGAAGGAAACAGAGACACAACT		57.2		
XPS2452F	9	TGTCTTTCGAATTTACATGTGT	XPS2452R	54.3	SL4.0ch09:65479794..65479953,65479840	160bp/117bp
XPS2452R	9	AGCGATGAACATAGAAACGA		54.9		
XPS2462F	9	CCCATAGTAATTGAGCATGTAAGG	XPS2462R	59.0	SL4.0ch09:65920134..65920268,65920143	213bp/350bp
XPS2462R	9	CAGCAACAATAATCGGCAAA		59.7		
XPS2465F	9	CGGGATGAGTTATTCCACCA	XPS2465R	60.7	SL4.0ch09:64998774..64998949,64998858	174bp/112bp
XPS2465R	9	GGAGTCGTTTGGTGTGAGGT		60.0		
XPS2466F	9	TTGGGTAAACTTTCACATATTCACA	XPS2466R	59.7	SL4.0ch09:67499665..67499745,67499665	163bp/117bp
XPS2466R	9	TTCAACTCACTTATTCCACACCA		59.5		
XPS2481F	9	CATTGACAGTTGATTTGGCTTT	XPS2481R	59.1	SL4.0ch09:60289700..60290300,60289953	212bp/342bp
XPS2481R	9	TCGAGTCCTTGCAGAGTTCTT		59.2		
XPS2483F	9	TGTGACGAAGCGTGTTTGA	XPS2483R	60.0	SL4.0ch09:65308300..65308999,65308603	250bp/166bp
XPS2483R	9	ACTCGATGCTTGATATTTGTATTG		57.5		
XPS2487F	9	GGTCGCATTTGTTGATACGATAC	XPS2487R	60.6	SL4.0ch09:64638298..64638870,64638584	180bp/147bp
XPS2487R	9	CGCCACGTGCACATAAAA		60.3		

XPS2495F	9	TGATTCGTGGAAAGTGACAAA	XPS2495R	59.2	SL4.0ch09:64641996	441bp/100+339bp, cut by HinFI
XPS2495R	9	AAAATGGAGAGGGGAGTGGT		59.8		
XPS2496F	9	AGGAAGGTTGGGAAGTTGCT	XPS2496R	60.1	SL4.0ch09:64646502	299bp/73+226bp, Cut by StyI
XPS2496R	9	TCTGGCTGTCCGTTCTTCTT		60.0		
XPS2515F	9	GCGTAATTCGTTCAGGTAATTTTT	XPS2515R	59.8	SL4.0ch09:65395877..65396106,65395992	143bp/197bp
XPS2515R	9	GTAGTTTTGGGTGAGCTGGTTT		59.6		
